# Clonal overlap and convergent clustering of T cell receptor signatures in Crohn’s disease in monozygotic twins

**DOI:** 10.1093/ibd/izag078

**Published:** 2026-06-05

**Authors:** Eelco C Brand, Romi Vandoren, Lisanne Lutter, Vincent M L Van Deuren, Nila H Servaas, Aridaman Pandit, Pieter Meysman, Bas Oldenburg, Femke van Wijk, Dutch TWIN-IBD consortium and the Dutch Initiative on Crohn and Colitis, Bas Oldenburg, Femke van Wijk, Eelco C Brand, Pieter Honkoop, Rutger J Jacobs, Bart L M Müskens, Cyriel Y Ponsioen, Nanne K H de Boer, Yasser A Alderlieste, Margot A van Herwaarden, Sebastiaan A C van Tuyl, Maurice W Lutgens, C Janneke van der Woude, Wout G M Mares, Daan B de Koning, Joukje H Bosman, Juda Vecht, Anneke M P de Schryver, Andrea E van der, Meulen-de Jong, Marieke J Pierik, Paul J Boekema, Robert J Verburg, Bindia Jharap, Gonneke Willemsen, Dorret I Boomsma, Jeroen M Jansen, Pieter C F Stokkers, Frank Hoentjen, Rutger Quispel, Carmen S Horjus Talabur Horje, Paul C van de Meeberg, Nofel Mahmmod, Meike M C Hirdes, Rachel L West, Marleen Willems, Itta M Minderhoud, Herma H Fidder, Fiona D M van Schaik, Nynke A Boontje, Sarah L Dijkstra, A Sophie Bakker, Rinse K Weersma, Marielle J L Romberg-Camps

**Affiliations:** Department of Gastroenterology and Hepatology, University Medical Center Utrecht, Utrecht University, Utrecht, 3584 CX, the Netherlands; Center for Translational Immunology, University Medical Center Utrecht, Utrecht University, Utrecht, 3584 CX, the Netherlands; Adrem Data Lab, Department of Computer Science, University of Antwerp, Antwerp, 2020, Belgium; Antwerp Unit for Data Analysis and Computation in Immunology and Sequencing, Antwerp, 2020, Belgium; Department of Gastroenterology and Hepatology, University Medical Center Utrecht, Utrecht University, Utrecht, 3584 CX, the Netherlands; Center for Translational Immunology, University Medical Center Utrecht, Utrecht University, Utrecht, 3584 CX, the Netherlands; Adrem Data Lab, Department of Computer Science, University of Antwerp, Antwerp, 2020, Belgium; Antwerp Unit for Data Analysis and Computation in Immunology and Sequencing, Antwerp, 2020, Belgium; T Cell Differentiation Lab, Department of Research, Sanquin Blood Supply Foundation, Amsterdam, 1066 CX, the Netherlands; Landsteiner Laboratory, Amsterdam Institute for Infection and Immunity, Cancer Center Amsterdam, Amsterdam UMC, University of Amsterdam, Amsterdam, 1105 AZ, the Netherlands; Oncode Institute, Utrecht, the Netherlands; Immunology Discovery Research, AbbVie Cambridge Research Center, Cambridge, MA, United States; Adrem Data Lab, Department of Computer Science, University of Antwerp, Antwerp, 2020, Belgium; Antwerp Unit for Data Analysis and Computation in Immunology and Sequencing, Antwerp, 2020, Belgium; Department of Gastroenterology and Hepatology, University Medical Center Utrecht, Utrecht University, Utrecht, 3584 CX, the Netherlands; Center for Translational Immunology, University Medical Center Utrecht, Utrecht University, Utrecht, 3584 CX, the Netherlands

**Keywords:** inflammatory bowel disease, IBD, immunology, T cell, pathogenesis

## Abstract

**Introduction:**

The dysregulated immune response in Crohn’s disease might result from disturbances in the T cell receptor (TCR) repertoire. To investigate this hypothesis, we compared the peripheral TCR repertoire within T cell subsets in twin pairs concordant and discordant for Crohn’s disease.

**Methods:**

We performed TCRα and TCRβ sequencing on peripheral flow-sorted CD4+ memory gut-homing (integrin α4β7), non–gut-homing (integrin α4β7−), and regulatory T cells (Tregs) from Dutch monozygotic Crohn’s disease concordant twins (n = 8), monozygotic Crohn’s disease discordant twins (n = 8), and healthy control subjects (n = 4). TCR diversity and clonality, overlap between individuals, convergence and enrichment, and clustering of the TCR repertoire was studied and compared with previously reportd Crohn’s disease–related TCRs.

**Results:**

Overall diversity and clonality was comparable between Crohn’s disease patients, healthy cotwins, and healthy control subjects. Comparing T cell subsets, a decreased diversity and increased clonality was observed for Tregs. Concordant Crohn’s disease twins had an increased overlap in TCRs for Tregs and CD4+ memory gut-homing T cells. Using TCR convergence, enrichment, and subsequent clustering analyses, we identified 8 clusters of TCRs potentially related with Crohn’s disease. The identified Crohn’s disease–related TCR signatures have not previously been described in relation to Crohn’s disease and thus far have mostly unknown antigen specificity.

**Conclusions:**

Increased overlap in the TCR repertoires of monozygotic twin pairs concordant for Crohn’s disease suggest that (antigen-driven) skewing of the TCR repertoire could play a role in the pathophysiology of Crohn’s disease. The identified TCR-based Crohn’s disease signatures are prime targets for further study into the pathophysiology of Crohn’s disease.

Key Messages **What is already known?** 

T cell receptor (TCR) repertoire disturbances may play a role in Crohn’s disease’s pathophysiology.

**What is new here?** 

In this study in monozygotic twin pairs discordant and concordant for Crohn’s disease, we identified 8 novel Crohn’s disease–related TCR signatures and found increased TCR overlap in concordant twin pairs, suggesting a role for (antigen-driven) skewing of the TCR repertoire in Crohn’s disease’s pathophysiology.

**How can this study help patient care?** 

A better understanding of the pathophysiology of inflammatory bowel disease can aid optimization of treatment and (immune) monitoring; the identified TCR signatures and TCR repertoire skewing are therefore targets for further investigation, including identification of the cognate antigens.

## Introduction

At the intestinal mucosa, the immune system discriminates between antigens originating from commensal and pathogenic microbiota, balancing immune response and tolerance. In the setting of Crohn’s disease (CD) the homeostasis is disrupted, leading to an inappropriate and persistent inflammation.^1^ The cause of this disturbance is currently poorly understood. A diverse T cell receptor (TCR) repertoire, consisting of a wide variety of unique TCR sequences across different T cell subsets, is needed to discriminate self from non-self and to defend the body from pathogens.^2^ In several immune-mediated diseases (eg, rheumatoid arthritis, psoriatic arthritis, systemic lupus erythematosus, and immunodeficiency disorders), disturbances of the TCR repertoire such as differences in TCR diversity and clonal expansion have been found.^3^^,^^4^

Previous studies into the TCR repertoire in CD reported lower diversity and increased oligoclonality (ie, presence of hyperexpanded T cell clones) in the peripheral blood and/or intestinal mucosa of patients with CD.^5–7^ In nonresponders to therapy, more within-patient overlap in the mucosal TCR repertoire before and after induction therapy with adalimumab, infliximab, or budesonide has been described.^5^ Postoperative recurrence after ileocecal resection has also been linked to more high-frequency mucosal T cell clones and lower TCR diversity at the moment of surgery, and more overlap between the TCR repertoire at the moment of surgery and 6 months thereafter.^8^ Preliminary data on the intestinal mucosal TCR repertoire in therapy-refractory CD showed a reduction in mucosal T cell hyperexpansion following hemopoietic stem cell transplantation in responders,^9^ although another stem cell transplantation study in CD contradicted these findings.^10^ A less diverse, more oligoclonal TCR repertoire with specific TCR clonotypes might thus play a role in the pathogenesis of CD, suggesting shared antigenic drivers or convergent T cell responses within CD.

The presence of dominant T cell clones raises the question what the cognate antigen(s) for these TCRs might be. Linking newly identified antigens to these TCRs could reveal important clues regarding the underlying disease mechanisms. Indeed, efforts have been made to identify CD-associated clonotypes. The aforementioned study on ileocecal resection did not identify specific shared ileal TCR clonotypes, probably due to the high variation in TCRs between patients.^8^ Nonetheless, a different approach focusing on the TCRα and TCRβ repertoire in peripheral blood identified a TCRα CDR3 motif that was more often found in CD patients than healthy control subjects or ulcerative colitis patients.^11^^,^^12^ T cells expressing these TCRα clonotypes had a gene expression profile similar to mucosa-associated invariant T cells and natural killer T cells and were coined Crohn’s disease–associated invariant T cells (CAITs). Interestingly, TCRβ clonotypes associated with these CAITs were highly variable between patients.^11^ Via a different approach, more than 1000 TCRβ-clonotypes potentially linked to CD were recently identified by analyzing the bulk TCRβ repertoire of healthy control subjects and CD and ulcerative colitis patients.^7^ However, none of these TCRβ clonotypes overlapped with the TCRβ repertoire of the earlier described CAITs.^7^^,^^11^

Studying the TCR repertoire in CD concordant and discordant monozygotic twin pairs can be a highly informative approach for identifying disease-related TCR clonotypes because the human leukocyte antigen (HLA) is shared between monozygotic twins, as are genetic and (childhood) environmental factors. Previous studies in healthy monozygotic twins have indeed shown an increase in the shared TCR sequences in peripheral blood both for CD4+ and CD8+ T cells.^13^^,^^14^ If a TCR sequence is shared more frequently among concordant twin pairs, this suggests a stronger association between that TCR and the disease, bringing the identification of CD-related TCR sequences within reach. An earlier study into the bulk blood TCR repertoire in twins with inflammatory bowel disease (IBD) including a small number of CD patients identified a few clonotypes potentially associated with IBD but did not specifically look at the CD-associated clonotypes.^15^ In addition, most studies have analyzed the TCR repertoire of total T cells, whereas focusing on specific T cell subsets may offer greater resolution in identifying CD–associated TCRs. In particular, potentially gut-homing (integrin α4β7+) T cells and regulatory T cells (Tregs) are of interest.

In this study, we analyzed the peripheral TCR repertoire in a unique cohort of monozygotic twins concordant or discordant for CD, aiming to identify shared clones as well as enriched or convergent TCRs beyond simple sequence overlap. Of note, most studies in CD focused on exact TCR clonotype matching between patients, while it is known that not only exact but also similar TCR sequences might recognize the same cognate antigen.^16^ By clustering TCR sequences, we expect to gain better insight into CD-specific TCR motifs. To provide additional biological context, analyses were performed within defined T cell subsets, including memory CD4+ gut homing (integrin α4β7+), memory CD4+ non–gut homing (integrin α4β7−), and CD4+ Tregs.

## Methods

### Study population

We included twin pairs from the ongoing prospective longitudinal Dutch TWIN-IBD (Twin cohort for the study of (pre)clinical inflammatory bowel disease in the Netherlands) study (Dutch Trial Register: NL6187).^17^ For the present cross-sectional study, we included (1) CD concordant monozygotic twin pairs (ie, both twins of the twin pair are affected by CD) and (2) CD discordant monozygotic twin pairs (ie, one of the twins of the twin pair is affected by CD). Twins affected by CD from CD concordant and discordant twin pairs are referred to as CD twins and individuals who are not diagnosed with IBD from CD discordant twin pairs are referred to as healthy cotwins. Unrelated individuals not affected by IBD, referred to as healthy control subjects, were recruited via the Mini Donor Service at the University Medical Center Utrecht.

### Ethical considerations

The research was carried out in accordance with the declaration of Helsinki and the Dutch Medical Research Involving Human Subjects Act. The TWIN-IBD study was approved by the medical ethics committee of the University Medical Center Utrecht (NL61114.041.17). All participants provided informed consent.

### Blood sampling, processing, and TCR sequencing

#### Sample collection and peripheral blood mononuclear cell isolation

Peripheral blood was collected from all participants in sodium heparin tubes. Peripheral blood mononuclear cells (PBMCs) were isolated using Ficoll Isopaque density gradient centrifugation (GE Healthcare Bio-Sciences) and were subsequently gradually frozen at −80 °C in RPMI 1640 medium containing 1% penicillin/streptomycin (Gibco), 1% L-glutamine (Thermo Fisher Scientific, Life Technologies), 20% fetal calf serum (FCS) (Invitrogen), and 10% dimethyl sulfoxide (Sigma-Aldrich) using a Corning CoolCell container (Sigma-Aldrich). The PBMCs were thereafter stored in liquid nitrogen until further use.

#### Flow cytometry and cell sorting

PBMCs were thawed and resuspended in RPMI 1640 medium containing 1% penicillin/streptomycin, 1% L-glutamine, and 10% FCS. PBMCs were first incubated with fixable viability dye eFluor506 (1:1000; eBioscience) in phosphate-buffered saline for 20 minutes at 4 °C and washed in fluorescence-activated cell sorting (FACS) buffer (cold phosphate-buffered saline with 2% FCS and 0.1% sodium-azide [Severn Biotech Ltd.]). For surface staining, cells were subsequently incubated for 20 minutes at 4 °C in FACS buffer containing 2% mouse serum (Bioconnect) and 8% Brilliant Stain Buffer (BD Biosciences) with antibodies directed against human CD3, CD4, CD8α, CD25, CD45RA, CD127, CCR7, integrin α4, integrin β7, and CCR9, and viability dye (Table S1). Alive CD3+CD8−CD4+CD25+CD127low (Tregs), CD3+CD8−CD4+CD25−CCR7+/−CD45RA−Integrinα4β7+ (CD4 gut-homing memory T cells), CD3+CD8−CD4+CD25−CCR7+/−CD45RA −Integrinα4β7− (CD4 non–gut-homing memory T cells) cells were sorted (Figure S1). The gut-homing and non–gut-homing CD45RA− CD4 T cell populations may contain, in addition to memory cells, a proportion of recently activated T cells. Between 45 000 and 100 000 cells were sorted per subset. Sorted T cell subsets were lysed in 450 µL TRIzol LS Reagent (Invitrogen) and stored at −80 °C until RNA isolation.

To check for FOXP3 (forkhead box P3) expression of the sorted Treg populations, a subsample of sorted Tregs and non-Tregs (alive CD3+CD8−CD4+CD25−) were fixed and permeabilized using 1 part fixation/permeabilization concentrate and 3 parts fixation/permeabilization diluent (eBioscience) for 30 minutes at 4 °C and subsequently stained for 30 minutes at 4 °C in 10× diluted Permeabilization Buffer (eBioscience) containing 1% rat serum (Thermo Fisher Scientific) and an anti-human FOXP3 antibody (Table S1 and Figure S1). Sorting and data acquisition were performed on a FACSAria III (BD) and analyzed using FlowJo Software v10 (Tree Star Inc.).

#### TCR sequencing

For total RNA extraction, after thawing at room temperature, 120 µL chloroform was added to the vials. The vials were shaken well, incubated at room temperature, and spun down at 12 000 *g* for 15 minutes at 4 °C. The aqueous phase was transferred into new vials. RNA was precipitated with 300 µL isopropanol in the presence of GlycoBlue (Invitrogen) and incubated for 1 hour at −20 °C. After spinning down at 12 000 *g* for 10 minutes at 4 °C, the supernatant was carefully discarded and the RNA pellet was washed twice with 562.5 µL 75% ethanol. The dried pellet was then dissolved in 15 µL RNase-free water and stored at −80 °C until quality control and library preparation. The purity, integrity and quantity of the isolated RNA was checked with the 2100 Bioanalyzer (Agilent) with Pico or Nano kit as appropriate and the Qubit (Invitrogen) according to the manufacturers’ protocol.

Nine microliters of the isolated RNA in RNase-free water was used per sample to construct TCR receptor repertoire libraries using the SMARTer Human TCR a/b Profiling Kit v2 (Takara bio), a unique molecular identifier (UMI)–based 5′ RACE (5′ rapid amplification of complementary DNA ends)–like method using switching mechanism at 5′ end of RNA Template (SMART) full-length complementary DNA (cDNA) synthesis technology, according to the manufacturer’s protocol. By incorporating UMIs, we were able to correct for potential polymerase chain reaction (PCR) bias after sequencing. In short, reverse transcription was performed with a TCR dT primer and further extended with nontemplated nucleotides, to which the TCR SMART UMI oligo anneal, using MMLV-derived SMARTScribe reverse transcriptase. Next, the cDNA was amplified in two semi-nested PCR steps. In the first PCR step, TRA and TRB constant region reverse primers and a human TCR universal forward primer were used. In the second PCR step, the PCR1 amplicons were targeted with reverse and forward primers including adapter and unique dual index sequences, enabling sample barcoding. NucleoMag Next-Generation Sequencing Clean-up and Size Select beads were used to purify the PCR products.

Before sequencing, pools were made containing libraries of 24 samples per pool, and each pool was purified aiming to enrich for 200- to 1000-bp-long cDNA. The pools were then sequenced twice on an Illumina NovaSeq6000 platform (Genomescan) to generate at least 10 million 150-bp-long paired-end reads per sample.

#### TCR sequencing data processing

The quality of the sequence results was checked with the multiQC toolkit. FASTQ raw data files were processed with Cogent NGS Immune Profiler Software version 1.0 (Takara Bio), which incorporates MIGEC version 1.2.9 and MiXCR version 2.1.8.^18^^,^^19^ We set identical molecular identifier group thresholds for all samples: 6 for TCRα and 3 for TCRβ.

#### Analyses

MiXCR output was combined across all patients, and nonproductive TCRs, containing nonstandard amino acids or unproductive V- and J-genes, were removed. Unique clonotypes are defined as a unique combination of V-, (D-), and J-fragments per patient and per cell type. Unique clonotypes were used to determine repertoire richness as well as Pielou evenness, which is a normalized Shannon diversity, using scikit-bio alpha diversity (v0.5.9; Python v3.8.18; Python Software Foundation). Richness and Pielou evenness were determined per chain type (TCRα and TCRβ) and patient group (CD twins, healthy cotwins, healthy control subjects). Exact overlap as well as the adjusted Morisita-Horn index were used to quantify repertoire overlap between concordant twins, discordant twins, unrelated CD pairs (ie, CD twins matched to an unrelated CD patient) and unrelated healthy pairs (ie, healthy cotwins and healthy control subjects). In contrast to the original Morisita-Horn index, this adjusted version emphasizes both on the presence and relative frequency of shared clonotypes, and reduces the effect of private nonoverlapping clones, using the following formula implemented in Python:


$$\mathrm{Adjusted}\;\mathrm{Morisita}-\mathrm{Horn}=\frac{2\sum_{i=1}^{P}N_{1i}N_{2i}}{(\sum_{i=1}^{P}f_{i}+\;\sum_{i=1}^{P}g_{i})*\;n_{1}*\;n_{2}}$$

With P representing all shared, public clonotypes and N_1i_ and N_2i_ representing the absolute abundance of shared clone i in both repertoires. The clonal relative frequency in both repertoires is given as f_i_ and g_i_, summed across all shared clones. Finally, n1 and n2 represent the total abundance per repertoire. Differences in overlap between the 4 groups was calculated using the Kruskal-Wallis test, followed up by post hoc pairwise Dunn’s test if there was a significant group difference and Bonferroni multiple testing correction.

TRIASSIC (v0.2.0; Python v3.10.14)^20^ was used to identify TCRs that were more convergent in the CD group compared with healthy cotwins and healthy control subjects. TRIASSIC compares clonotypes across the full cohort and quantifies convergence of a TCR by counting clonotypes that are similar but developed independently. These events of convergent recombination were identified by the presence of (1) the same clonotype in a different individual, (2) a clonotype with differing nucleotide sequence coding for the same amino acid sequence regardless of individual, and (3) a highly similar clonotype (TCRdist^16^^,^^21^ score ≤12.5), regardless of the individual. The TCRdist threshold corresponds with approximately 1 amino acid sequence difference in TCR sequence, allowing for the identification of TCRs that potentially recognizes the same antigen and thereby provides biologically meaningful results. For each TCR, we identified the number of convergence events within its 2048 most similar clonotypes, as identified by TCRdist. A 2-tailed Fisher exact test was used to calculate enrichment in the number of convergent events originating from one group or another. Clonotypes that were statistically significantly associated with CD (enrichment > 0, *P* value ≤.05) were selected. To reduce potential effects of pre-existing/preclinical CD patterns within the healthy cotwins (who are at increased risk of developing CD), convergent TCRs that were only found when comparing against the healthy cotwin group and not healthy control subjects were excluded. We checked for potential effects of age, sex, and thiopurine and anti-tumor necrosis factor α use by fitting a model in the convergent analyses to estimate the effect size of these factors on the convergence values and visually assess changes in the density of convergent TCRs. Clonotypes that are significantly convergent in CD were then clustered based on sequence similarity (Hamming distance = 1) using ClusTCR (v1.0.2)^22^ across the full cohort. Features of these CD convergent clusters, such as sample distribution, clone counts, cell type distribution and epitope annotation, were extracted for the most promising clusters. Epitope specificity per clonotype was predicted using the ImmuneWatch Detect tool^23^ at the default confidence level 0.2.

Significantly neighbor enriched (SNE) TCRs (defined by TCRdist unit radius ≤18.5);^24^ were identified using a synthetic TCR background (10× size of original repertoire). The TCRdist unit radius is recommended to be set at 18.5 to enable to detect TCRs with 1 to 2 amino acid sequence differences, enabling the capture of local clusters within an individual’s TCR repertoire. This was generated by random breaking, shuffling, and re-pairing of VDJ fragments of the clonotypes within the repertoire. Based on the background, expected neighbor counts were calculated and compared with the actual observed neighbors per repertoire and per chain type. Enrichment of these SNE TCRs was calculated using SciPy’s (v1.8.0) hypergeometric survival function and adjusted for multiple testing using the Bonferroni correction. Further prioritization was applied by selecting those TCR clusters that were significantly convergent for CD and that contained TCR sequences that were SNE TCRs.

Last, we compared all unique TCRs in the present study with TCRs potentially associated with CD as reported in the literature. To this end, we compared all the unique TCRs, the unique TCRs within the CD patients specifically, and the TCRs identified in our clustering analysis with the TCRs identified in literature. This includes the study of Rosati et al^15^ in twins concordant and discordant for IBD, the study of Rosati et al,^11^ which identified CAITs in their dataset, and the study of Rios Martini et al,^25^ which reported an extensive list of yeast-reactive TCRs in the setting of CD. Finally, we aimed to compare against the “enhanced TCR sequences” associated with CD as described by Pesesky et al,^7^ but unfortunately the TCR sequences could not be shared by the authors, and therefore we compared with a patent^26^ based on earlier analyses of the same group, which included 2010 unique TCRs.

## Results

### Baseline characteristics

**Figure 1:**
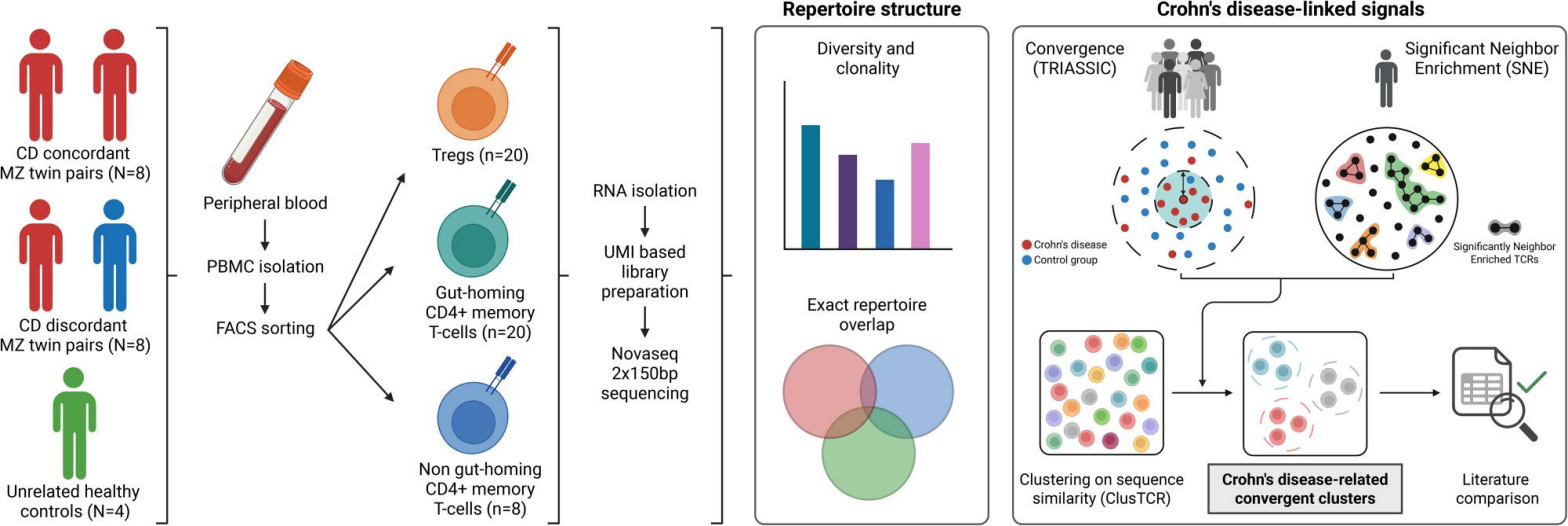
Study overview. From peripheral blood mononuclear cells (PBMCs) of 4 monozygotic twin pairs concordant for Crohn’s disease (CD), 4 monozygotic (MZ) twin pairs discordant for CD, and 4 unrelated healthy control subjects, regulatory T cells (Tregs) (alive CD3+CD4+CD25+CD127low), gut-homing CD4+ memory T cells (alive CD3+CD8−CD4+CD25−CCR7+/−CD45RA−Integrinα4β7+), and non–gut homing CD4+ memory T cells (alive CD3+CD8−CD4+CD25−CCR7+/−CD45RA−Integrinα4β7−) were fluorescence-activated cell sorting sorted (Figure S1). RNA was isolated and used to prepare a unique molecular identifier (UMI)–based T cell receptor (TCR) library for 2 × 150 bp sequencing on a NovaSeq platform. From this, TCRα and TCRβ repertoire analyses were performed. Diversity, clonality and repertoire overlap was studied. Subsequently, TCR convergence analyses followed by clustering, and TCR significant neighbor enrichment analyses followed by clustering were performed. Both methods identify not only exact amino acid matches, but also similar sequence matches against a TCR background, across the whole cohort or within a single repertoire, respectively. As a final step, we combined the results of both analyses to identify the most promising CD-related TCR clusters and compared the identified CD-related clusters to the existing literature. This figure has been created with www.biorender.com.

TCR sequencing was performed for 4 CD concordant monozygotic twin pairs (8 individuals), 4 CD discordant monozygotic twin pairs (8 individuals), and 4 healthy control subjects (Table 1, Figure 1;  Figure S2). TCR repertoire data were generated for all 20 individuals for Tregs and CD4 gut-homing memory T cells, and for 1 CD concordant twin pair, 2 CD discordant twin pairs, and 2 healthy control subjects for CD4 non–gut-homing memory T cells.

**Table 1:** Baseline and sample characteristics of included patients for TCR sequencing.

	Crohn’s disease concordant twin pairs (4 pairs, 8 individuals)	Crohn’s disease discordant twin pairs (4 pairs, 8 individuals)	Healthy control subjects (4 individuals)
**Demographics and clinical characteristics**			
Female	2 (25)	4 (50)	2 (50)
Age, y	24.5 (22.5-26.8)	35 (30-41.8)	36 (32.8-40)
Body mass index, kg/m^2^	22.3 (20.7-22.6)	23.1 (21-25.3)	NA^a^
Current smoking	1 (12.5)	2 (25)	NA^a^
Disease phenotype			
Crohn’s disease	8 (100)	4 (50)	0
No inflammatory bowel disease	0	4 (50)	4 (100)
Zygosity			
Monozygotic	8 (100)	8 (100)	—
Dizygotic	0	0	—
**Crohn’s disease characteristics^b^**			
Disease duration, mo	70.9 (10.1-142)	104 (17.4-189)	—
Age of diagnosis (Montreal classification)			
A1 (≤16 y)	2 (25)	0	—
A2 (17-39 y)	6 (75)	3 (75)	—
A3 (≥40 y)	0	1 (25)	—
Location (Montreal classification)			
L1 (ileum only)	4 (50)	2 (50)	—
L2 (colon only)	1 (12.5)	1 (25)	—
L3 (ileocolonic)	3 (37.5)	1 (25)	—
L4 (proximal of ileum)	2 (25)	0	—
Behavior (Montreal classification)			
B1 (nonstricturing, nonpenetrating)	2 (25)	3 (75)	—
B2 (stricturing)	4 (50)	1 (25)	—
B3 (penetrating)	2 (25)^c^	0	—
p (perianal modifier)	0	0	—
Patient Harvey-Bradshaw index^d^	2.5 (2-3.8)	3 (1-5)	—
Signs of disease activity^e^	4 (50)	2 (50)	—
Current Crohn’s disease medication			
No Crohn’s disease medication	0	1 (25)	—
5-Aminosalicylic acid	0	1 (25)	—
Corticosteroids	0	0	—
Methotrexate	0	0	—
Thiopurine	7 (87.5)	3 (75)	—
Anti-tumor necrosis factor α	5 (62.5)	0	—
Vedolizumab (anti-integrin α4β7)	0	0	—
Ustekinumab (anti-interleukin-12/23)	0	0	—
**Sample characteristics**			
Regulatory T cells^f^			
Number of samples for TCR sequencing	8 (100)	8 (100)	4 (100)
Number of cells per sample	94 100 (78 700-100 000)	100 000 (72 900-100 000)	99 000 (89 500-100 000)
Number of million sequence reads	10.8 (9.9-14.4)	17.4 (15.6-20.8)	14.5 (12.5-16.5)
CD4 gut-homing memory T cells^f^			
Number of samples for TCR sequencing	8 (100)	8 (100)	4 (100)
Number of cells per sample	83 100 (57 800-100 000)	100 000 (91 500-100 000)	100 000 (92 400-100 000)
Number of million sequence reads	13.7 (10.2-17.7)	17.8 (15.7-19.9)	12.8 (12.0-13.2)
CD4 non–gut-homing memory T cells^f^			
Number of samples for TCR sequencing	2 (25)	4 (50)	2 (50)
Number of cells per sample^g^	100 000-100 000	100 000 (100 000-100 000)	100 000-100 000
Number of million sequence reads^g^	10.1-15.2	18.2 (16.0-19.2)	11.3-21.0

Values are n (%), median (Q1-Q3), or range.

Abbreviations: NA, not applicable; TCR, T cell receptor.

a Data were not collected for healthy control subjects.

b Crohn’s disease characteristics are only relevant to the Crohn’s disease patients; therefore, proportions reflect the percentage relative to the total number of Crohn’s disease patients per group.

c One of these patients is classified as B2 and B3.

d The patient-Harvey-Bradshaw index^27^ is a symptom-based score with higher scores indicating more active disease.

e Disease activity was defined as Harvey Bradshaw index >4 or signs of inflammation as noted during rectoscopy.

f Regulatory T cells: alive CD3+CD8−CD4+CD25+CD127low cells; CD4 gut-homing memory T cells: alive CD3+CD8−CD4+CD25−CCR7+/−CD45RA−Integrin α4β7+ cells; CD4 non–gut-homing memory T cells: alive CD3+CD8−CD4+CD25−CCR7+/−CD45RA−Integrin α4β7− cells.

g If only 2 samples were used for a cell type, we here report the minimum and maximum value.

Concordant CD twin pairs were younger than discordant CD twin pairs and healthy control subjects (median ages 24.5, 35, and 36, respectively). The CD patients within the concordant twin pairs compared with the CD patients from discordant twin pairs were often younger at diagnosis (Montreal classification A1: 25% vs 0%) and had more often stricturing and penetrating disease (Montreal classification B2: 50% vs 25%; Montreal classification B3: 25% vs 0%). The concordant and discordant CD twins were comparable regarding the patient Harvey-Bradshaw Index (median 2.5 vs 3), proportion of patients with signs of disease activity (50% vs 50%), and use of thiopurines (87.5% vs 75%), although only CD patients from concordant twin pairs used anti-tumor necrosis factor α (62.5%). After a median follow-up of 7 years following sampling, none of the healthy cotwins from CD discordant twin pairs developed clinically overt CD.

No statistically significant difference in the proportion of Tregs, gut-homing CD4+ memory T cells, or non–gut-homing CD4+ memory T cells was found comparing the 16 CD twins, 4 healthy cotwins, and 6 healthy control subjects for whom flow cytometry data were available (Table S2 and Figure S3).

We generated a median of 15.1 million (Q1-Q3: 12.1-18.5 million) sequence reads per sample containing median 54.8% (Q1-Q3: 49.7%-56.9%) TCRα, median 43.9% (Q1-Q3: 41.9%-48.7%) TCRβ, and median 1.1% (Q1-Q3: 0.9%-1.5%) undetermined reads. After UMI correction, a median number of 13 400 (Q1-Q3: 6530-24 800) TCRα and 20 400 (Q1-Q3: 11 300–40 700) TCRβ clonotypes remained.

### Richness, diversity, and clonal expansion

**Figure 2:**
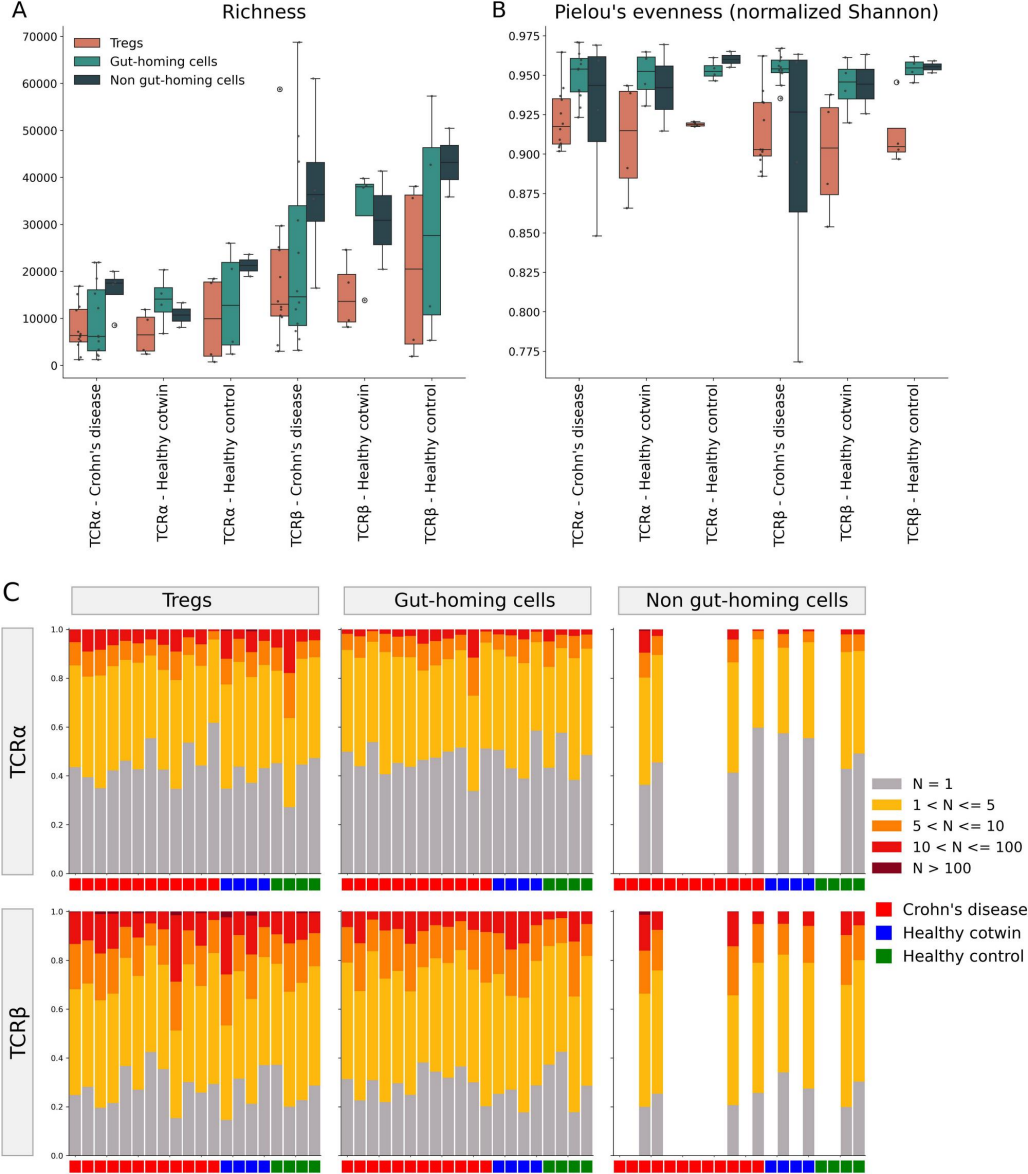
Richness, diversity, and clonal expansion. (A) Richness (ie, the number of unique T cell receptor [TCR] sequences) and (B) diversity (expressed as Pielou evenness, which is a normalized Shannon metric) are displayed per cell type, separately for TCRα and TCRβ sequences, subdivided for Crohn’s disease patients, healthy cotwins, and unrelated healthy control subjects. Overall, the richness and diversity was lower for regulatory T cells (Tregs) compared with gut-homing and non–gut-homing CD4+ memory T cells, irrespective of disease subtype. (C) Absolute clonal expansion (ie, the number of T cells per unique TCR) per cell type, separately for TCRα and TCRβ sequences, is displayed per individual. Especially in the TCRβ sequences, clonal expansion of the Tregs is noted.

No clear difference was found in richness (ie, unique TCR sequences), diversity (Pielou’s evenness), and clonal expansion in the TCRα and TCRβ repertoire between CD patients, healthy cotwins, and healthy control subjects (Figure 2). The diversity in the TCRα and TCRβ repertoires of Tregs was numerically lower compared with the TCR repertoires of gut-homing CD4+ memory T cells and non–gut-homing CD4+ memory T cells, irrespective of disease state. In line with this, the TCRβ repertoire, and to a lesser extent the TCRα repertoire from Tregs, was more clonally expanded (Figure 2).

### Overlap in TCR sequences

**Figure 3:**
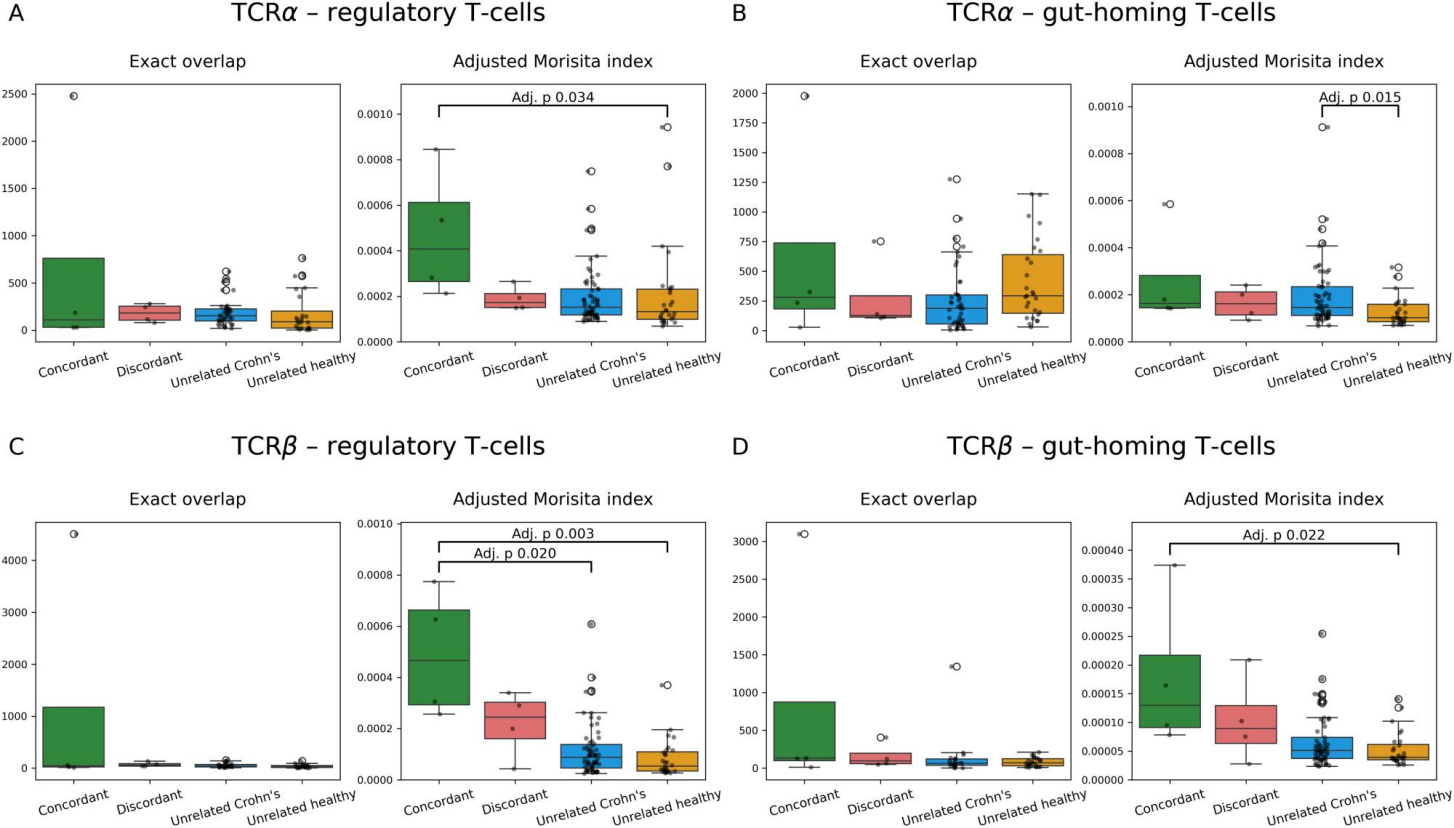
Overlap between individuals in T cell receptor (TCR) sequences. Exact clonotype overlap and the adjusted Morisita-Horn index is displayed within Crohn’s disease concordant twin pairs, Crohn’s disease discordant twin pairs, pairs of unrelated Crohn’s disease patients, and pairs of unrelated healthy participants (ie, healthy cotwins and healthy control subjects). Results are separately displayed for (A, B) TCRα and (C, D) TCRβ for (A, C) regulatory T cells (Tregs) and (B, D) gut-homing CD4+ memory T cells. There is a statistically significantly (Bonferroni adjusted *P* ≤ .05) increased overlap within the TCR repertoire within concordant Crohn’s disease twin pairs compared with unrelated healthy control subjects for Tregs and gut-homing CD4+ memory T cells within the TCRβ repertoire, and for Tregs in the TCRα repertoire.

We hypothesized that if the TCR repertoire is associated with CD, a higher overlap within concordant CD twin pairs could be expected compared with discordant CD twin pairs and pairs of unrelated individuals. For TCRβ sequences, a statistically significant (Bonferroni-adjusted *P* ≤ .05) higher overlap (expressed as the adjusted Morisita-Horn index, taking size of the different repertoires into account) was noted within the concordant CD pairs compared with pairs of unrelated healthy individuals (ie, healthy cotwins and healthy control subjects) for both Tregs and gut-homing CD4+ memory T cells (Figure 3). Overall, there was a numerical trend toward lower overlap within discordant CD twin pairs. No overlap analyses were performed for the non–gut-homing CD4+ memory T cells because TCR repertoires for these cell types were only determined in a subgroup of participants. For the TCRα repertoire, only for Tregs was an increase found in the adjusted Morisita-Horn index comparing concordant CD twin pairs with unrelated pairs of healthy individuals. These results suggest that based on overlap analyses there is skewing of the peripheral TCRβ repertoire of Tregs and gut-homing CD4+ memory T cells associated with CD.

### TCR convergence, enrichment, clustering, and comparison with existing literature

To further explore which TCRs might be associated with CD, we took two complementary approaches, namely TCR convergence analyses followed by clustering and TCR neighbor enrichment analyses followed by clustering. Both methods identify not only exact amino acid matches, but also similar sequence matches against a TCR background, across the whole cohort or within a single repertoire, respectively. As a final step, we combined the results of both analyses to identify the most promising CD-related TCR clusters.

**Figure 4:**
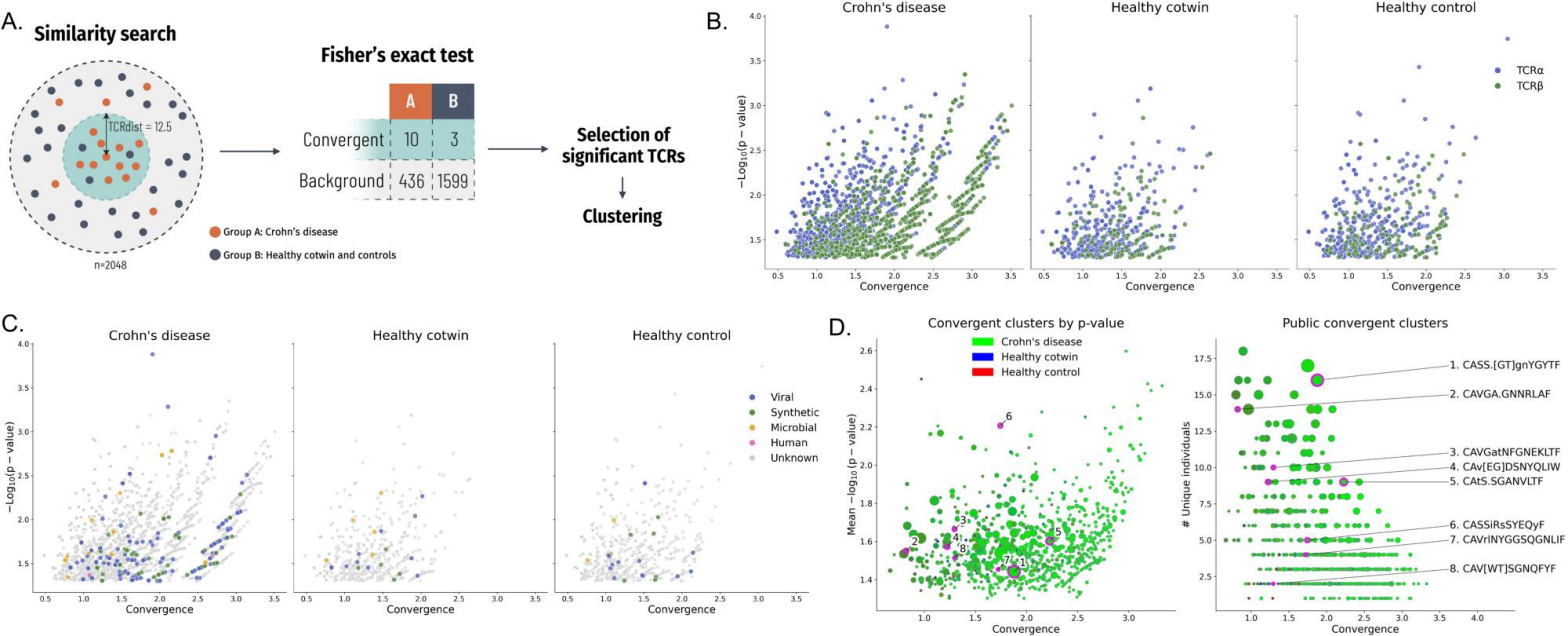
T cell receptor (TCR) convergence and potentially Crohn’s disease (CD)–related TCR clusters. (A) Schematic representation of how TCR convergence is calculated. Within a group (eg, CD patients), TCRs that are similar based on a TCRdist of 12.5 are counted and compared with the number of TCRs found in the comparator groups. (B, C) The statistically significant (*P* ≤ .05) TCRs and their convergence measure are displayed separately for CD patients, healthy cotwins, and healthy control subjects. The TCRs are colored by (B) TCRα and TCRβ sequences and (C) the predicted epitope recognition based on ImmuneWatch Detect.^24^ (B) The TCR convergence in general is larger for TCRβ sequences and is more pronounced in CD patients. (C) The vast majority does not have a prediction on the epitope specificity, with potential viral antigens being the largest group of assigned potential targets of the TCRs. (D) The TCRs with statistically significant convergence within the CD group were clustered based on sequence similarity using ClusTCR. This shows multiple clusters that have increased convergence in more than 1 CD sample, left on *P* value, right on number of CD samples with that cluster. Taking statistical significance and number of participants in whom a TCR related to a cluster was found into account, combined with the TCR neighbor enrichment analyses (Figure 5;  Figure S6), 8 potentially CD-related clusters (Table 2;  Table S3) could be identified and are highlighted in the plot.

In TCR convergence analyses, for each TCR the number of convergence events (ie, the same or similar clonotypes that developed independently either in a different individual, a different nucleotide sequence, or within a TCRdist distance ≤12.5), is calculated and compared among (1) CD patients, (2) healthy cotwins, and (3) healthy control subjects. TCRs that showed a higher number of convergence events in CD patients compared with healthy samples, potentially indicating a shared driver for convergence, were identified using enrichment testing based on the Fisher exact test. The analysis revealed 8123 convergent TCRs when comparing the CD patients with all healthy individuals. A larger number of convergent TCRs was found in the CD group than within the healthy cotwins and healthy control subjects, suggesting a potential CD-related skewing toward higher convergence in these samples. Among the convergent TCRs, there was no preference for a specific cell type (Figure 4A, B; Figure S4), and for most of these TCRs ImmuneWatch Detect was unable to predict their epitope specificity against known microbial, viral, or self-antigens (Figure 4C). Adjustment for age, sex, thiopurine, and anti-tumor necrosis factor α use only had a marginal effect on the TCR convergence results. The observed convergence patterns remained mainly associated with disease status (ie, CD, healthy cotwin, or healthy control) (Figure S5). In order to identify groups of similar TCRs found across different CD patients, the significantly convergent TCRs were clustered based on sequence similarity using ClusTCR. This resulted in a large number of potentially CD-related clusters that varied in size and patient distribution. Each cluster now contained multiple distinct but CD-convergent TCRs, grouped based on potential recognition of shared antigens that might play a role in CD.

To further explore the relevant convergent clusters, we first performed TCR enrichment analyses using significantly enriched neighbors. In this analysis, the number of neighbor TCR sequences (TCRdist distance ≤18.5) within the repertoire of one individual is compared with a synthetic background. This allows for the identification of TCRs that have more similar TCRs than expected by chance within a single repertoire.

**Figure 5:**
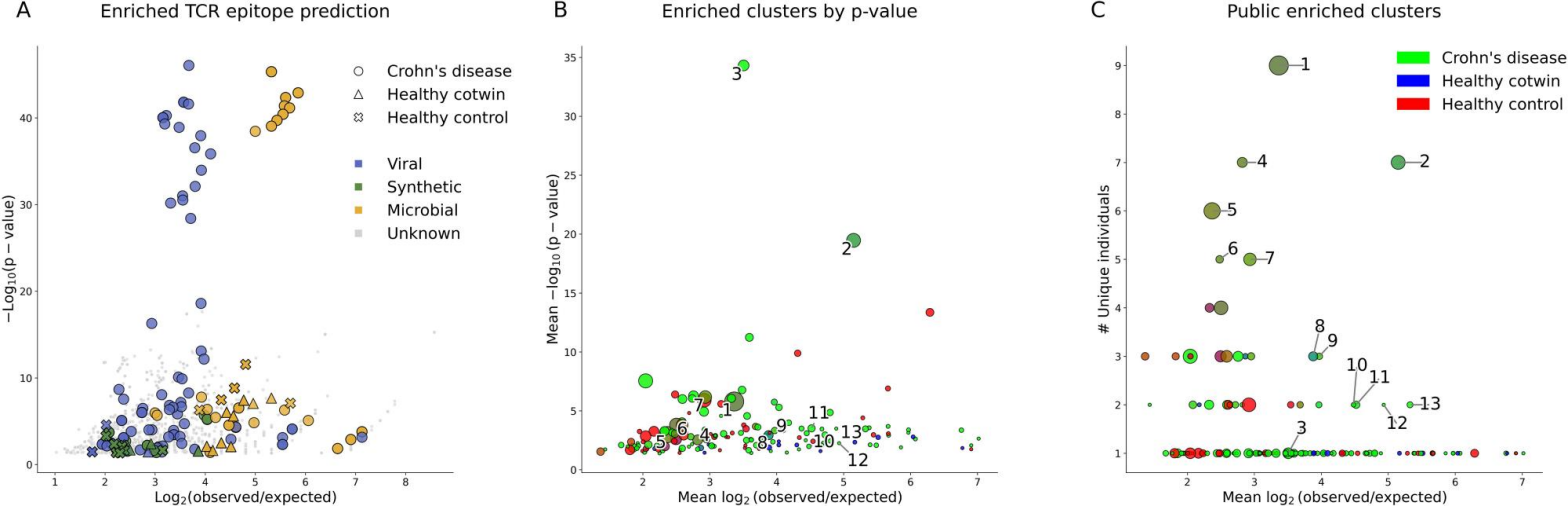
T cell receptor (TCR) neighbor enrichment analyses and clustering. TCR neighbor enrichment is calculated comparing the number of neighbor TCR sequences (ie, similar TCR sequences) within 1 TCR repertoire compared with a synthetic background repertoire. (A) Only statistically significantly (Bonferroni-adjusted *P* value ≤ .05) enriched TCRs are displayed. Shapes refer to whether the TCR is found in Crohn’s disease patients, healthy cotwins, or healthy control subjects. Colors show the predicted epitope based on ImmuneWatch Detect.^24^ Within Crohn’s disease patients, a few TCRs of non–gut-homing CD4+ memory T cells that are partly predicted to be directed against viral and partly against microbial antigens are the most statistically significantly enriched TCRs. The results are displayed in more detail for TCRα and TCRβ, cell type, antigen prediction, and individual participant subdivided for Crohn’s disease patients, healthy cotwins, and healthy control subjects in Figure S6. (B, C) Clustering based on sequence similarity (ClusTCR, Hamming distance = 1)^22^ was performed on the neighbor-enriched TCRs. These cluster plots display the mean enrichment score, mean *P* value, and the number of unique patients in which TCRs from a cluster are encountered. The color gradient indicates the proportion of TCRs found across the different types of participants. Based on either exhibiting a highly significant *P* value or high convergence across individuals, 13 promising clusters were selected that are mainly encountered in Crohn’s disease patients (Table S3).

These analyses revealed different TCRs that were more enriched within individual CD patients than within healthy cotwins and healthy control subjects. The most statistically significant TCRs for CD patients were TCRα sequences predicted to be directed against microbiota, likely mucosa-associated invariant T cells based on their V- and J-gene usage (TRAV1-2 combined with TRAJ12, TRAJ20, or TRAJ33). Additionally, TCRβ sequences predicted to be directed against the influenza A virus were also enriched in CD patients (Figure 5A;  Figure S6A–C). Interestingly, the most statistically significantly enriched TCRs were found in the repertoire of one individual (Figure S6D). Subsequently, these enriched TCRs were also clustered based on sequence similarity across the different samples, reflecting cohort-wide enrichment patterns. This resulted in a total of 13 enriched clusters that seem to be of interest due to their enrichment in mainly CD patients and clusters being shared among multiple individuals (Figure 5B, C).

These 13 neighbor-enriched clusters were then used to further prioritize the list of convergent clusters from the previous analysis. Specifically, it was determined which CD-convergent clusters also contained TCRs present in any of the 13 neighbor-enriched clusters. Convergent clusters that showed this dual signal, both convergence and neighbor enrichment, were considered as the most promising CD-related clusters. This integrative approach resulted in 8 prioritized convergent clusters (Table 2; Table S3 and Figure S7) spanning both TCRα and TCRβ chains, which may represent CD-associated TCR patterns. It remains to be determined toward which antigens the majority of these TCRs are directed.

**Table 2:** Overview of the 8 selected CD-related TCR clusters.

Cluster number	Cluster motif	TCR chain	Unique TCRs	Unique participants	Unique CD patients	TCRs identified in CD patients (%)	Tregs (%)	Non–gut-homing T cells (%)	Gut homing T cells (%)	Antigen prediction (%)^a^	Predicted antigen^a^
1	CASS.[GT]gnYGYTF	TCRβ	42	16	10	87.7	35.4	24.6	40	0	—
2	CAVGA.GNNRLAF	TCRα	7	14	9	62.8	39.5	20.9	39.5	0	—
3	CAVGatNFGNEKLTF	TCRα	5	10	6	70.8	35.3	23.5	41.2	0	—
4	CAv[EG]DSNYQLIW	TCRα	7	9	6	66.7	33.3	26.7	40	73.3	Bacteria
5	CAtS.SGANVLTF	TCRβ	16	9	7	91.7	20.8	4.2	75	0	—
6	CASSiRsSYEQyF	TCRβ	6	5	3	83.3	33.3	50	16.7	100	Influenza A virus
7	CAVrlNYGGSQGNLIF	TCRα	3	4	2	60	60	20	20	0	—
8	CAV[WT]SGNQFYF	TCRα	2	2	2	100	100	0	0	0	—

The cluster motifs, TCR chain, number of unique TCRs included per cluster, the number of all participants and of CD patients specifically in which TCRs belonging to a cluster are identified, the percentage of T cells of a cluster that were identified in CD patients, the proportion of TCRs in a cluster per T cell subtype, the proportion of TCRs per cluster for which an antigen could confidently be predicted, and the predicted antigen are shown for the 8 selected CD-related T cell receptor clusters. The 8 selected clusters represent TCR clusters that were significantly convergent for CD and that contained TCR sequences that were significantly neighbor-enriched TCRs. A detailed overview of the 8 CD-related TCR clusters can be found in Table S3. Non–gut-homing T cells are alive CD3+CD8−CD4+CD25−CCR7+/−CD45RA−Integrin α4β7− T cells. Gut-homing T cells are alive CD3+CD8−CD4+CD25−CCR7+/−CD45RA−Integrinα4β7+ T cells. Tregs are alive CD3+CD4+CD8−CD25+CD127low T cells.

Abbreviations: CD, Crohn’s disease; TCR, T cell receptor; Treg, regulatory T cell.

a Antigen prediction is performed with ImmuneWatch Detect tool^24^ with a confident prediction set at the default confidence level 0.2.

### Comparison with the literature

In order to validate earlier reported potentially CD-related TCRs from literature, we compared these TCRs with (1) all TCRs in our cohort, (2) all TCRs within the CD patients in our cohort, (3) all convergent clusters, and (4) the selection of 8 CD-related clusters from the present study (Table 3). These comparisons were made against the suggested CD-related TCRs as reported by Rosati et al,^11^^,^^15^ Rios Martini et al,^25^ and a published patent.^26^

**Table 3:** Comparison of TCRs in present study with CD-associated TCR sequences identified in literature.

Reference dataset	Clonotypes in reference dataset	Compared with all TCRs		Compared with all TCRs in CD patients		Compared with all TCRs identified in the convergent cluster analyses		Compared with 8 selected clusters	
		Unique clonotypes	Clonotype count	Unique clonotypes	Clonotype count	Unique clonotypes	Clonotype count	Unique clonotypes	Clonotype count
**TCRα^a^**									
Rosati et al 2020^15^ predicted TCRs	8	3 (37.5)	12	2 (25)	3	0	0	0	0
Rosati et al 2020^15^ viral TCRs^b^	4	4 (100)	79	4 (100)	44	1 (25)	14	1 (25)	14
Rosati et al 2022^11^ CAIT cells	33	7 (21.2)	14	6 (18.2)	10	0	0	0	0
Rios Martini et al 2023^25^	19 457	5505 (28.3)	18 216	4173 (21.4)	9761	214 (1.1)	986	2 (0.01)	23
Patent for CD TCRs^26^^,^^c^	NA	NA	NA	NA	NA	NA	NA	NA	NA
**TCRβ^a^**									
Rosati et al 2020^15^ predicted TCRs	20	5 (25)	10	4 (20)	7	0	0	0	0
Rosati et al 2020^15^ viral TCRs^b^	3	3 (100)	69	3 (100)	39	2 (66.7)	66	0	0
Rosati et al 2022^11^ CAIT cells	43	1 (2.3)	1	0	0	0	0	0	0
Rios Martini et al 2023^25^	20 899	1160 (5.6)	1832	749 (3.6)	1011	48 (0.2)	129	1 (0.005)	1
Patent for CD TCRs^26^^,^^c^	2010	133 (6.6)	160	99 (4.9)	108	2 (0.1)	2	0	0

Values are n (%), unless otherwise indicated. The numbers in this table are unique TCR sequences based on V-gene, CDR3, and J-gene sequences. An overview of all overlapping clonotypes can be found in Table S4.

Abbreviations: CAIT, Crohn’s disease–associated invariant T; CD, Crohn’s disease; NA, not applicable; TCR, T cell receptor.

a In the present study, there were 388 918 unique TCRα and 11 113 820 unique TCRβ sequences considered for this comparison.

b Full TCRs were not available in this reference dataset, so matches and unique sequences was only determined based on the CDR3 sequence and not the V- and J-gene information.

c Originally, we aimed to compare to the CD enhanced TCR sequences identified by Pesesky et al.^7^ Unfortunately, the list of these sequences could not be shared by the author. This patent includes a list of CD-associated TCRs from earlier analyses from the same group.

Of the TCR sequences reported in literature, 21.2% to 37.5% of the TCRα sequences and 2.3% to 25% for the TCRβ sequences were also present in our cohort (Table 3;  Table S4). When only comparing the TCR sequences from literature with the TCR sequences in CD patients in our cohort, the overlap was slightly lower (TCRα: 18.2%-25%, TCRβ: 2.9%-20%). When looking at our convergent cluster analyses, none of the reported CAIT TCR sequences^11^ were found. A small proportion of the reported yeast-reactive TCRs,^25^ including TCRs reactive to *Candida tropicalis* and *Saccharomyces cerevisiae*, were linked to the TCRs identified in our convergent cluster analyses (TCRα: 1.1%; TCRβ: 0.2%). Only 0.1% of the CD-related enhanced TCRβ sequences as reported in the patent^26^ was also identified in our convergent cluster analyses. The 8 selected CD-related clusters from the present study almost have no overlap with published TCRs.

In conclusion, a substantial proportion of potentially CD-related TCR sequences from literature were present in the TCR repertoires in the present study but were not specifically found to be CD enriched. The TCR sequences we identified through cluster analyses were mostly new (ie, not overlapping with the earlier reported TCRs).

## Discussion

Within the setting of monozygotic twin pairs concordant and discordant for CD, the richness, diversity, and clonal expansion of the TCRα and TCRβ repertoire of CD4+ memory gut-homing T cells, CD4+ non–gut-homing T cells, and Tregs was comparable between CD patients, healthy cotwins, and healthy control subjects. Across CD4+ memory gut-homing T cells, CD4+ memory non–gut-homing T cells, and Tregs, the TCR repertoire of Tregs was less diverse and more clonally expanded. Interestingly, we found an increase in clonotype overlap in the TCR repertoire of twin pairs concordant for CD but not for discordant twin pairs, which hints at skewing of the TCR repertoire within CD. Next, we identified potential CD-related TCR sequences and TCR clusters via TCR convergence and neighbor enrichment analyses. Most of these TCRs do not yet have known antigen specificity. The majority of TCRs within these CD-related clusters have not previously been linked to CD.

A less diverse and more oligoclonal repertoire in blood and mucosa of CD patients has been reported previously.^5–7^ Furthermore, in a mouse model it has been shown that an oligoclonal Treg repertoire is associated with spontaneous development of intestinal inflammation, which could be suppressed by the transfer of Tregs with a diverse repertoire.^28^ However, in our study the peripheral TCR diversity and clonality per CD4+ cell type was comparable among CD patients, healthy cotwins, and healthy control subjects.

Increased overlap in the TCR repertoire of monozygotic twin pairs is expected, based on their shared genetic background, including sharing of HLA alleles^29^ and environmental factors. Studies into the TCR repertoire of healthy monozygotic twin pairs showed increased overlap,^13^^,^^30^ although the TCR repertoire per cotwin still is person specific.^31^ In a previous study into the peripheral TCR repertoire among IBD-discordant and concordant twins, with a low number of CD concordant twins, no increase in overlap was found for CD concordant pairs compared with CD discordant pairs.^15^ This might partly be explained by the fact that healthy cotwins from CD discordant pairs are at increased risk of developing CD^32^ and might therefore already partly display CD-related features, although none of the healthy cotwins developed clinically overt CD during follow-up. We did find an increased overlap in the TCR repertoire within CD concordant twin pairs compared with random pairs of healthy unrelated individuals, pointing toward antigen-driven TCR repertoire skewing in CD.

Highly similar TCR sequences may recognize the same epitopes.^16^^,^^33^ Therefore, we not only focused on exact TCR clonotypes, but also performed convergence and enrichment analyses followed by clustering to identify potentially CD-related TCR patterns. Via this approach, we were able to identify 8 distinct clusters of TCRs. Interestingly, the TCR sequences in these enriched clusters are independent from reported CD-related TCR sequences^11^^,^^15^^,^^25^^,^^26^ such as CAITs. The low percentage of overlap with earlier reported sequences has recently also been noted by Pesesky et al^7^ when they compared their identified CD-related enhanced TCRβ-sequences with the CD-related TCRs from literature.^11^^,^^25^ This reflects the complexity of identifying CD-related specific TCRs and also warrants further exploration in independent cohorts of our and other identified CD-related TCRs to determine whether they represent potential disease drivers, epiphenomena, and/or reflections of shared immune history (eg, viral or microbial exposure).

Ultimately, the goal is to dissect the role of specific antigens and antigen-specific T cells in CD. Currently it is impossible to reliably predict the cognate antigen for each TCR in silico.^34^ However, by using the ImmuneWatch Detect tool^24^ we were able to predict antigen specificities for some of the identified 8 CD-related TCR clusters based on known epitopes, using existing literature and computational models, including microbial and influenza A antigens. Still, for most TCRs no antigen specificity could be confidently predicted. It is important to bear in mind that this tool (and other TCR antigen prediction tools) are biased toward viral rather than self epitopes, as the majority of TCR data have been generated in relation to viral infections or vaccination studies. This can also be the explanation why we and Rosati et al^15^ found TCRs directed against influenza A viruses to be associated with CD. Microbial antigens could be of particular interest in the pursuit of CD-driving antigens because alterations in the gut microbiome have been implicated in CD, with microbiome alterations observed not only in established disease,^35^ but also in treatment-naive CD patients,^36^ people at risk of developing IBD,^17^ and within the prediagnostic phase of CD.^37^ Studies into microbiota- and mycobiota-reactive T cells in the setting of CD showed that microbiota-reactive CD4+ T cells in CD are skewed toward a T helper 17 phenotype,^38^^,^^39^ while for mycobiota-reactive CD4+ T cells a T helper 1 skewed phenotype was found.^25^ Unfortunately, TCR sequencing data can currently not be directly linked to the microbial composition. Establishing such a connection would ultimately provide crucial insights into the immune-microbiome interactions underlying CD.

Our study has several strengths. By studying the TCR repertoire in monozygotic twins, we could compare HLA-identical individuals, with shared genetic and (childhood) environmental factors, reducing the influential factors on the TCR repertoire. We studied both the TCRα and TCRβ repertoire in different CD4+ T cell subsets, increasing the resolution compared with, for example, whole-blood sequencing. It has been shown that TCRs that are not identical, but are sufficiently similar can recognize the same antigen.^16^^,^^33^ By not only focusing on exact TCRs, but also including similarity-based convergence and neighbor enrichment analyses, we were able to identify potentially CD-related TCR clusters, rather than rely only on exact clonotype matches or enrichment.

Our study also has some limitations. A limited number of participants was included, which is partly compensated for by the twin design; however, a larger number of participants would have added larger statistical power. Due to the nature of overlap analyses, it was not possible to adjust for potential confounders such as age, sex, and medication use. However, covariate adjustment for the convergence results showed that these factors did not explain the observed convergence patterns. The sequencing technique used allows for the TCR sequencing of a large number (in our case, 100 000) of T cells per sample. However, it is impossible in bulk sequencing to pair TCRα and TCRβ sequences. A paired TCR would have enabled a more comprehensive and precise study of the complete TCR repertoire. Last, we chose to focus on peripheral CD4+ T cells, so no information on the CD8+ and the mucosal TCR repertoire in CD was generated.

In conclusion, the increased overlap in the peripheral TCR repertoire within CD-concordant monozygotic twin pairs points toward a potential role for (antigen-driven) skewing of the TCR repertoire in the pathophysiology of CD. We identified novel CD-related TCR clusters with mostly unknown cognate antigens that are prime targets for further study into the pathophysiology of CD.

## Supplementary Material

izag078_Supplementary_Data

## Data Availability

The data underlying this article will be shared on reasonable request to the corresponding author.
