# Supplementary material for: Clonal overlap and convergent clustering of T cell receptor signatures in Crohn’s disease in monozygotic twins
Source: Inflamm Bowel Dis. 2026 Jun 5;32(8):1561–75. doi: 10.1093/ibd/izag078 (PMC13414540; doi:10.1093/ibd/izag078)
Supplement: izag078_Supplementary_Data [file izag078_supplementary_data.zip › 2026.03.30 Supp figure 5 - TCR-seq TWIN-IBD.pdf]

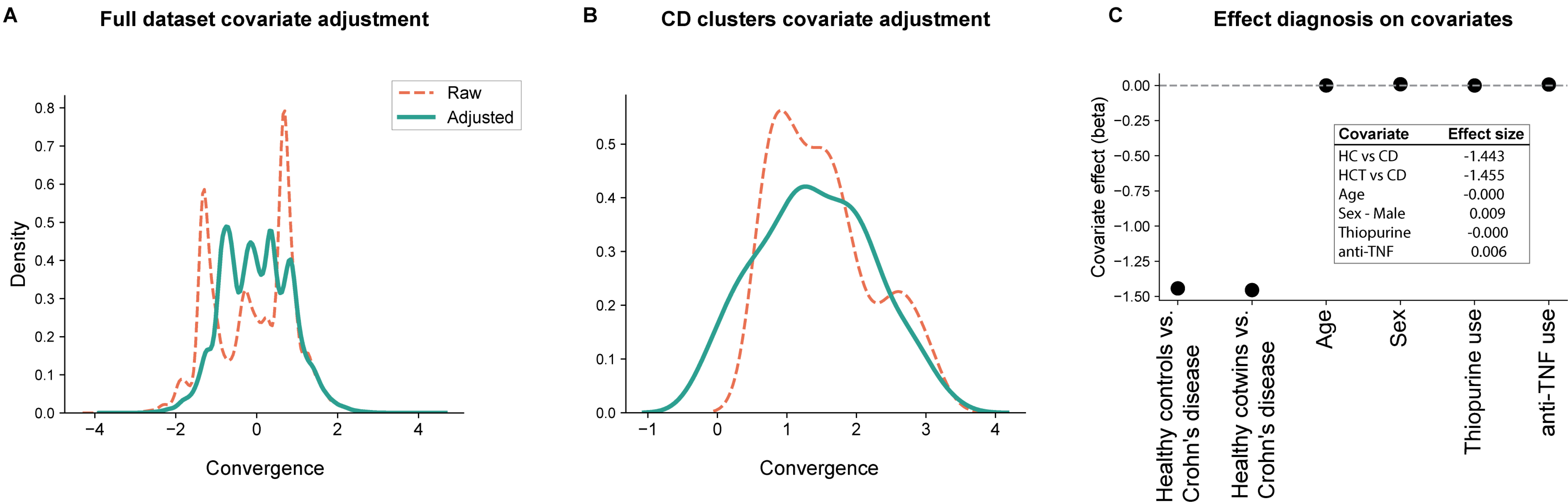
A
Full dataset covariate adjustment
Raw
Adjusted
Density
Convergence
B
CD clusters covariate adjustment
Convergence
C
Effect diagnosis on covariates
Covariate effect (beta)
Covariate
Effect size
HC vs CD
-1.443
HCT vs CD
-1.455
Age
-0.000
Sex - Male
0.009
Thiopurine
-0.000
anti-TNF
0.006
Healthy controls vs. Crohn's disease
Healthy cotwins vs. Crohn's disease
Age
Sex
Thiopurine use
anti-TNF use
