# Supplementary material for: Clonal overlap and convergent clustering of T cell receptor signatures in Crohn’s disease in monozygotic twins
Source: Inflamm Bowel Dis. 2026 Jun 5;32(8):1561–75. doi: 10.1093/ibd/izag078 (PMC13414540; doi:10.1093/ibd/izag078)
Supplement: izag078_Supplementary_Data [file izag078_supplementary_data.zip › 2026.03.30 Supp figure 7 - TCR-seq TWIN-IBD.pdf]

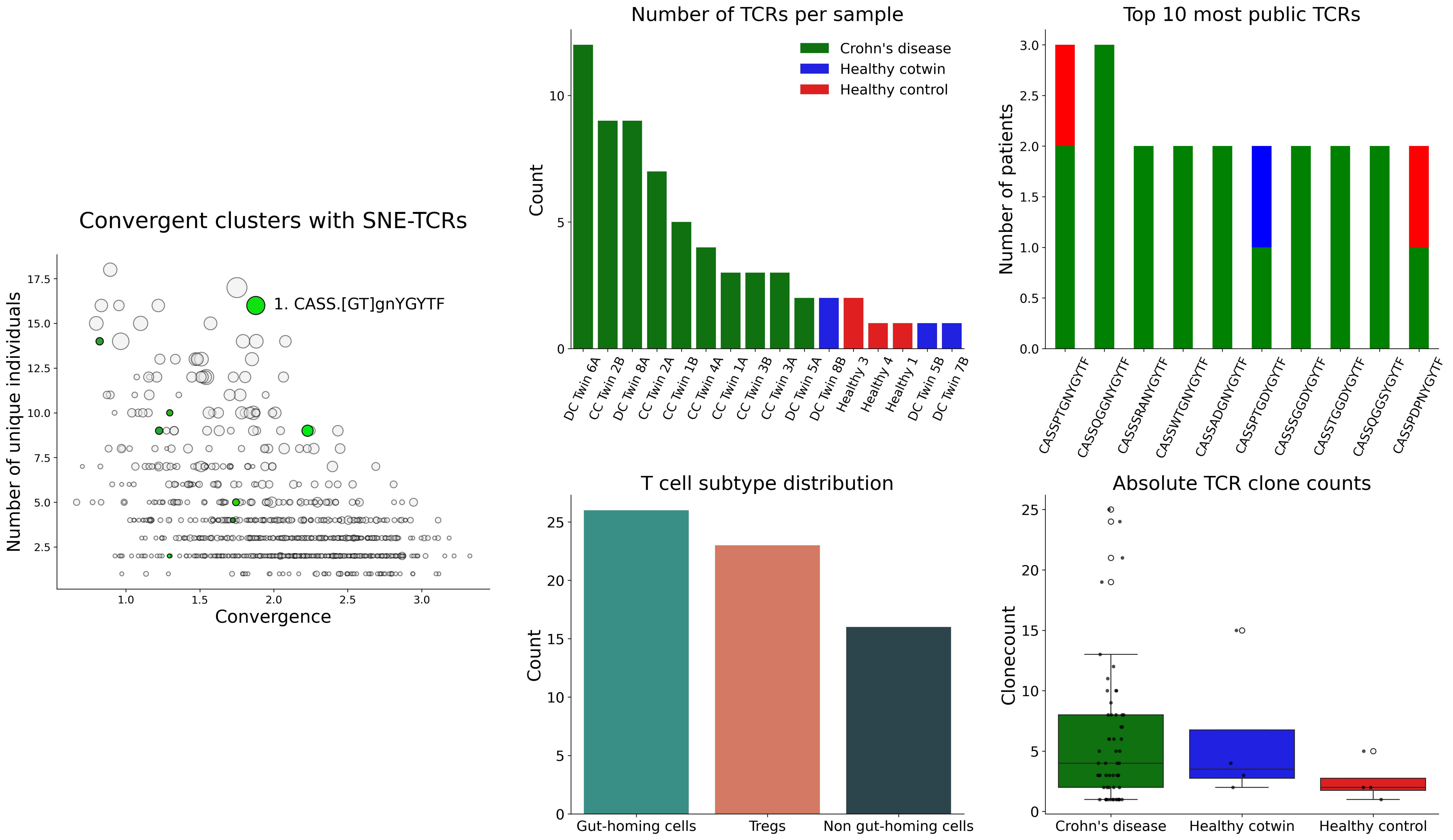
Convergent clusters with SNE-TCRs
Number of unique individuals
Convergence
1. CASS.[GT]gnYGYTF
Number of TCRs per sample
Count
Crohn's disease
Healthy cotwin
Healthy control
DC Twin 6A
CC Twin 2B
DC Twin 8A
CC Twin 2A
CC Twin 1B
CC Twin 4A
CC Twin 1A
CC Twin 3B
CC Twin 3A
DC Twin 5A
DC Twin 8B
Healthy 3
Healthy 4
Healthy 1
DC Twin 5B
DC Twin 7B
Top 10 most public TCRs
Number of patients
CASSPTGNYGYTF
CASSQGGNYGYTF
CASSSRANYGYTF
CASSWTGNYGYTF
CASSADGNYGYTF
CASSPTGDYGYTF
CASSSGGDYGYTF
CASSTGGDYGYTF
CASSQGGSYGYTF
CASSPDPNYGYTF
T cell subtype distribution
Count
Gut-homing cells
Tregs
Non gut-homing cells
Absolute TCR clone counts
Clonecount
Crohn's disease
Healthy cotwin
Healthy control
