# Supplementary material for: Clonal overlap and convergent clustering of T cell receptor signatures in Crohn’s disease in monozygotic twins
Source: Inflamm Bowel Dis. 2026 Jun 5;32(8):1561–75. doi: 10.1093/ibd/izag078 (PMC13414540; doi:10.1093/ibd/izag078)
Supplement: izag078_Supplementary_Data [file izag078_supplementary_data.zip › 2025.10.31 Supp figure 3 - TCR-seq TWIN-IBD - flow results.pdf]

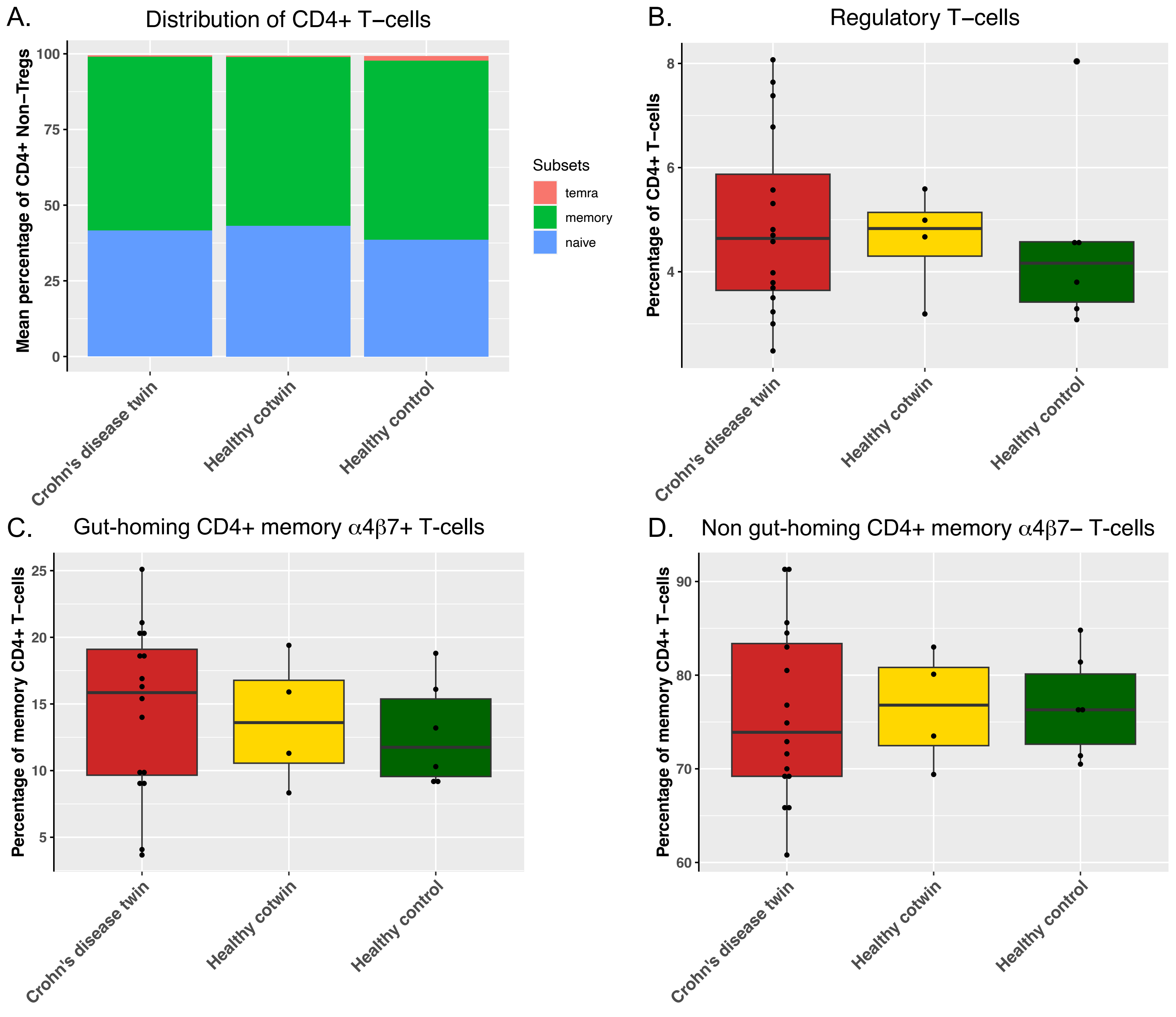
A.
Distribution of CD4+ T−cells
Mean percentage of CD4+ Non−Tregs
100
75
50
25
0
Subsets
temra
memory
naive
Crohn's disease twin
Healthy cotwin
Healthy control
B.
Regulatory T−cells
Percentage of CD4+ T−cells
8
6
4
Crohn's disease twin
Healthy cotwin
Healthy control
C.
Gut-homing CD4+ memory α4β7+ T-cells
Percentage of memory CD4+ T−cells
25
20
15
10
5
Crohn's disease twin
Healthy cotwin
Healthy control
D.
Non gut-homing CD4+ memory α4β7− T-cells
Percentage of memory CD4+ T−cells
90
80
70
60
Crohn's disease twin
Healthy cotwin
Healthy control
