# Supplementary figures and images for: Clonal overlap and convergent clustering of T cell receptor signatures in Crohn’s disease in monozygotic twins
Source: Inflamm Bowel Dis. 2026 Jun 5;32(8):1561–75. doi: 10.1093/ibd/izag078 (PMC13414540; doi:10.1093/ibd/izag078)

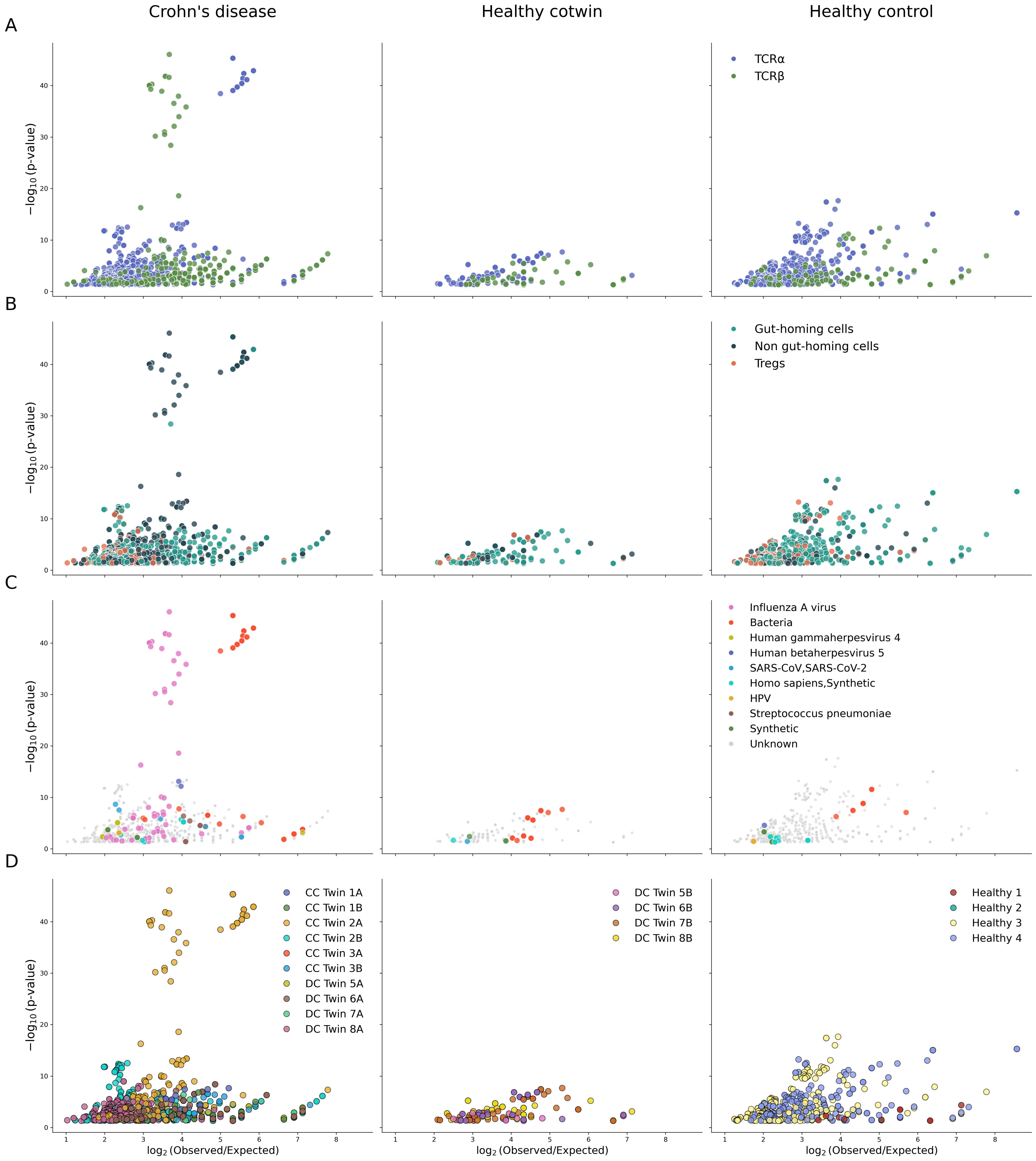

Supplement: izag078_Supplementary_Data [file izag078_supplementary_data.zip › 2026.03.30 Supp figure 6 - TCR-seq TWIN-IBD.pdf]

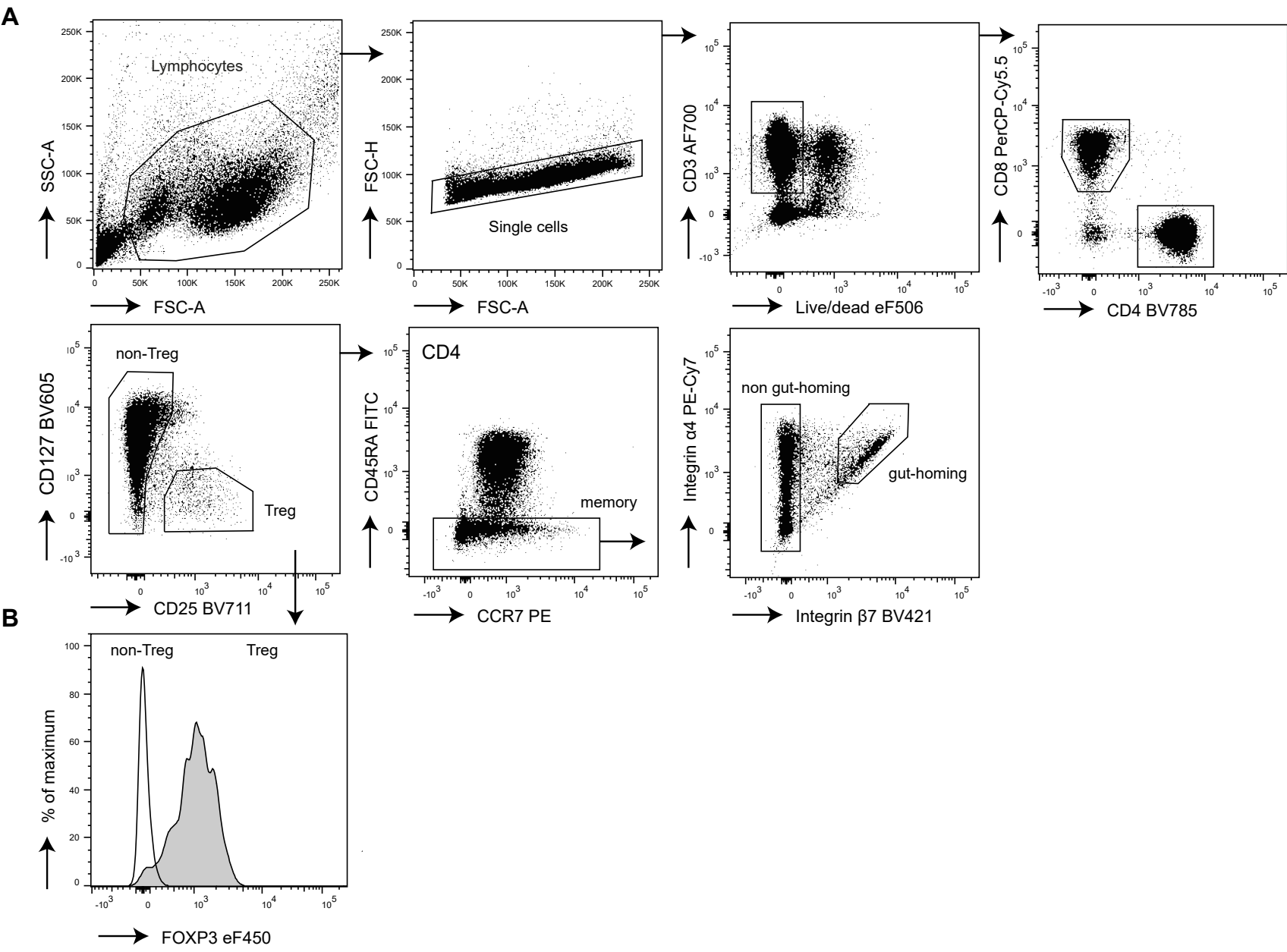

Supplement: izag078_Supplementary_Data [file izag078_supplementary_data.zip › 2025.10.31 Supp figure 1 - TCR-seq TWIN-IBD - sort strategy.pdf]

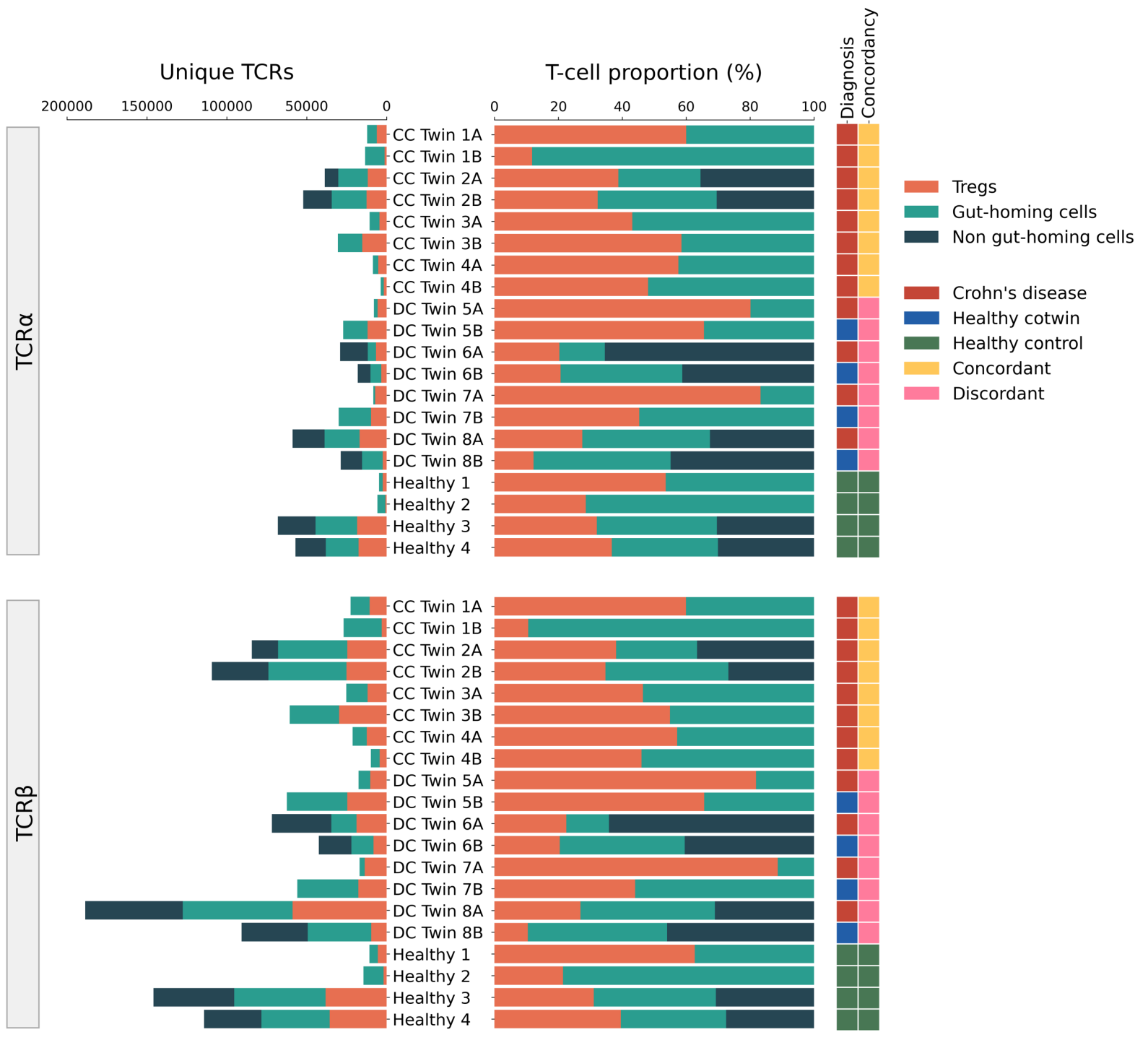

Supplement: izag078_Supplementary_Data [file izag078_supplementary_data.zip › 2025.10.31 Supp figure 2 - TCR-seq TWIN-IBD - sample outline.pdf]

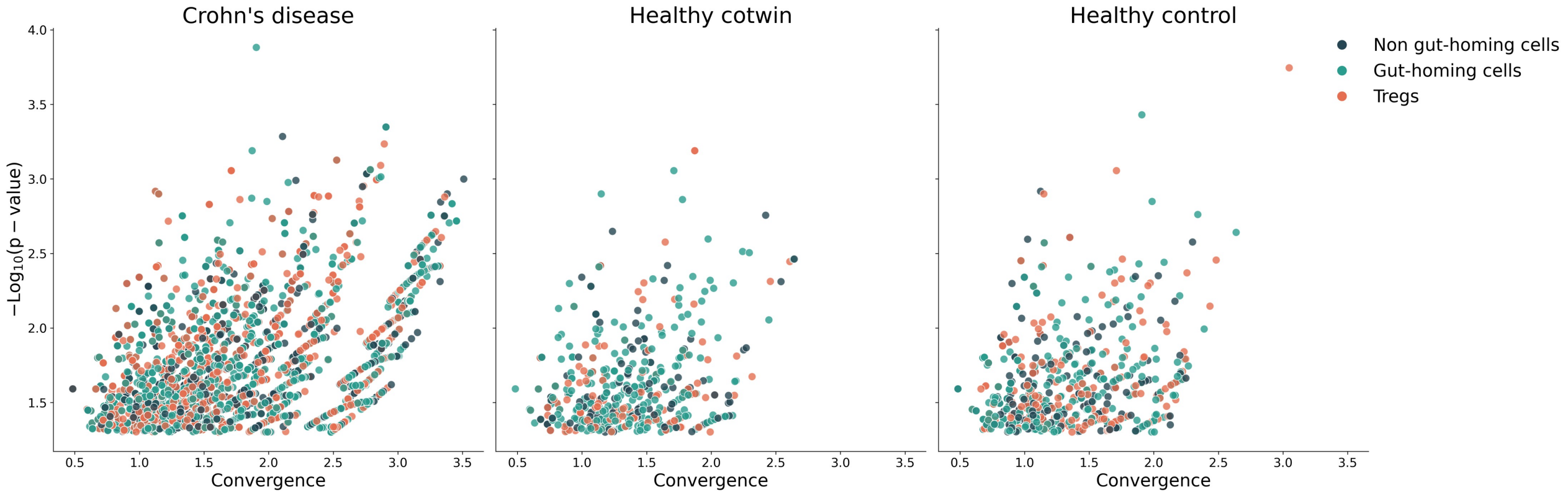

Supplement: izag078_Supplementary_Data [file izag078_supplementary_data.zip › 2025.10.31 Supp figure 4 - TCR-seq TWIN-IBD.pdf]
